# Semi-Interpenetrating Network Anion Exchange Membranes by Thiol–Ene Coupling Reaction for Alkaline Fuel Cells and Water Electrolyzers

**DOI:** 10.3390/molecules28145470

**Published:** 2023-07-17

**Authors:** Zhiyu Jin, Xiuyang Zou, Guodong Xu, Zhe Sun, Feng Yan

**Affiliations:** Jiangsu Engineering Laboratory of Novel Functional Polymeric Materials, Jiangsu Key Laboratory of Advanced Negative Carbon Technologies, Suzhou Key Laboratory of Soft Material and New Energy, College of Chemistry, Chemical Engineering and Materials Science, Soochow University, Suzhou 215123, China; 20204209321@stu.suda.edu.cn (Z.J.); 20204009045@stu.suda.edu.cn (X.Z.); xuguodong19@163.com (G.X.)

**Keywords:** anion exchange membranes, semi-interpenetrating network, fuel cells, water electrolyzers

## Abstract

In this work, a thiol–ene coupling reaction was employed to prepare the semi-interpenetrating polymer network AEMs. The obtained QP-1/2 membrane exhibits high hydroxide conductivity (162.5 mS cm^−1^ at 80 °C) with a relatively lower swelling ratio, demonstrating its mechanical strength of 42 MPa. This membrane is noteworthy for its improved alkaline stability, as the semi-interpenetrating network effectively limits the attack of hydroxide. Even after being treated in 2 M NaOH at 80 °C for 600 h, 82.5% of the hydroxide conductivity is maintained. The H_2_/O_2_ fuel cell with QP-1/2 membrane displays a peak power density of 521 mW cm^−2^. Alkaline water electrolyzers based on QP-1/2 membrane demonstrated a current density of 1460 mA cm^−2^ at a cell voltage of 2.00 V using NiCoFe catalysts in the anode. All the results demonstrate that a semi-interpenetrating structure is a promising way to enhance the mechanical property, ionic conductivity, and alkaline stability of AEMs for the application of alkaline fuel cells and water electrolyzers.

## 1. Introduction

Global environmental and energy issues have been greatly exacerbated by the usage of fossil fuels. Currently, people are looking for sustainable and clean energy sources to replace traditional energy sources [1]. Hydrogen energy is a clean and renewable energy source. Fuel cells have promising potential in terms of generating electricity by using clean hydrogen energy [2,3,4]. Moreover, the electrolysis is a clean and efficient method for obtaining hydrogen from water [5,6,7]. The anion exchange membrane fuel cells (AEMFCs) and anion exchange membrane water electrolysis (AEMWE) have several advantages such as fast oxygen reduction reactions, inhibited fuel crossover, and low commercial cost [8,9]. Thus, AEMFCs and AEMWE technologies can be considered eco-friendly and sustainable alternatives to traditional energy sources that can help mitigate the problems caused by global environmental and energy issues [10].

The essential component of AEMFCs and AEMWE is the anion exchange membrane (AEM), which is designed for separating the cathode and anode of the battery, and it is crucial for ion transport [11]. It directly determines the performance of AEMFCs, including service life and power density [12]. Nevertheless, the need to strike a balance between the mechanical properties and ionic conductivity of AEMs presents an urgent challenge. Poly(arylene)-based polymer backbones prepared by an acid-catalyzed polycondensation are popular in the boom of ether-free polymers. The resulting ether-free polymer frameworks are remarkable for their chemical durability, as there are no alkaline-sensitive ether linkages. This offers a promising alternative to traditional AEM materials with ether-free backbones and facile synthetic procedures [13,14,15]. However, large-scale commercialization still faces some challenges, as quaternized poly(arylene piperidinium) (QAPAP) AEMs, particularly the quaternized poly(biphenyl piperidinium) (QAPBP) counterpart, often exhibit serious dimensional swelling which jeopardizes their mechanical properties and durability [16]. Insufficient alkaline stability is another significant problem faced by AEMs. AEMs made up of poly(phenylene oxide) (PPO), polysulfone (PSU), and polyether ketone (PEK) typically undergo notorious chain degradation under extreme pH conditions [17,18]. This is because high concentrations of OH^−^ tend to destroy the aryl ether (C-O) bonds within these polymers, causing damage to the long-term stability of AEMs [19]. The dilemma of balancing the properties of ion-conducting and mechanically supported polymers in the formation of AEMs can be solved through cross-linking to form a semi-interpenetrating polymer network [20,21]. The two polymers in this network can function independently, allowing the AEM to possess both efficient ion transport properties and excellent mechanical properties. Additionally, the semi-interpenetrating structure of the network prevents excessive swelling of the membrane, ultimately allowing efficient ion conduction under a comparatively low IEC [12].

In this work, semi-interpenetrating (sIPN) polymer network structures were constructed by the poly(aryl piperidinium) polymers (PAP-TP-85) and hypercrosslinked polymers (QC-DO). A series of anion exchange membranes were designed and prepared by adjusting the mass ratio between QC-DO and PAP-TP-85. The rigid PAP-TP-85 and flexible QC-DO were contained to weigh the contradiction between ionic conductivity and swelling performance. Moreover, the oxygen-containing hydrophilic polymer network ensures that the anion exchange membrane has a high hydration number, which contributes to the ionic conductivity of the membranes. Subsequently, the morphology of the composite membrane was characterized by SEM test, and the mechanical strength of the composite membranes was characterized by tensile strength and elongation at break. High ionic conductivity and good alkaline stability of composite membranes were observed. The AEMFC and AEMWE test demonstrated that the QP-1/x AEMs with the semi-interpenetrating network have high potential for AEMFC and AEMWE applications.

## 2. Results and Discussion

### 2.1. Synthesis and Structure Characterization

**Scheme 1:**
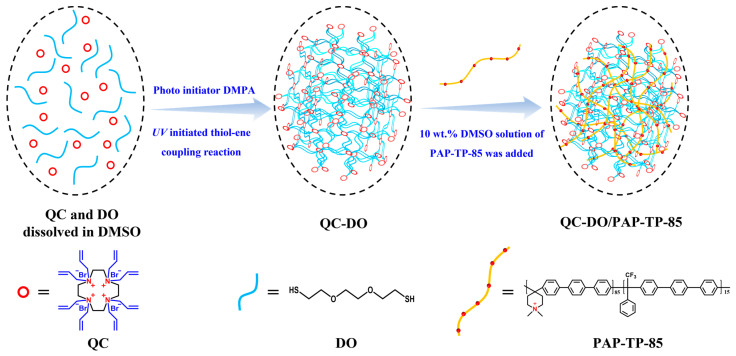
Preparation route for QP-1/x (1/x = 1:1, 1:2, 1:3) AEMs with semi-interpenetrating network.

The preparation route for QP-1/x (1/x = 1:1, 1:2, 1:3) AEMs with the semi-interpenetrating network is shown in Scheme 1. The successful preparation of QC, PAP-N, and PAP-TP-85 was determined using ^1^H NMR. The ^1^H NMR spectra of QC were obtained, as shown in Figure S1, and the ^1^H NMR spectra of PAP-N and PAP-TP-85 are displayed in Figure S2 and Figure S3, respectively. These spectra showed shifts from the arylene protons at 7–8 ppm, as well as shifts from the piperidinium ring and the methyl protons between 2.0 and 3.6 ppm. Additionally, protonation of the piperidine by TFA led to the splitting of signals from the resulting piperidinium protons [16]. The hypercrosslinked polymers QC-DO could not be dissolved in the deuterated reagent due to the occurrence of a cross-linking reaction. Thus, as shown in Figure S4, FT-IR spectroscopy was used to characterize QC-DO. The disappearance of the peak (*v* = 1639) in QC-DO after the cross-linking reaction provided strong evidence supporting the formation of a covalent cross-linking network [22].

### 2.2. Morphological Characterization of sIPN AEMs

**Figure 1:**
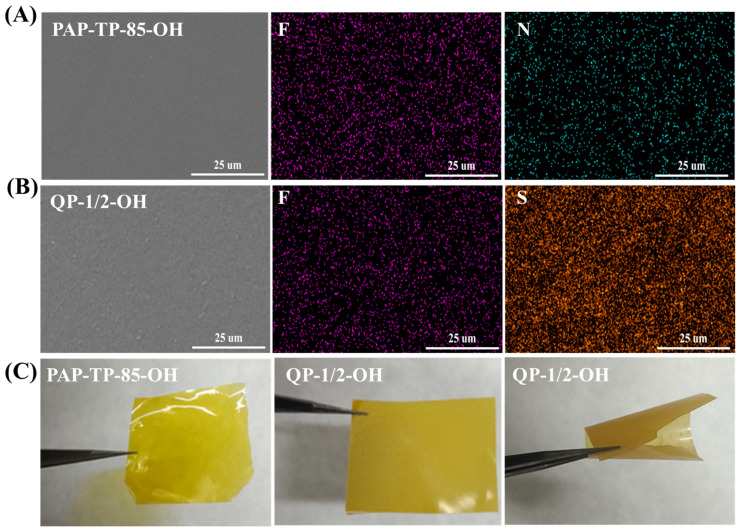
(**A**) SEM image of PAP-TP-85 AEM and corresponding EDS image of the distribution of F and N in it. (**B**) SEM image of QP-1/2 AEM and corresponding EDS image of the distribution of F and S in it. (**C**) The photo of PAP-TP-85 AEM and QP-1/2 AEM.

The microphase morphology of the PAP-TP-85 and QP-1/2 AEMs were evaluated using the SEM. Figure 1A,B showed that all membrane surfaces are smooth. As shown in Figure 1B, the energy dispersive spectroscopy (EDS) test verified the distribution of elemental sulfur in the QP-1/2 AEM, indicating the successful construction of the sIPN network. Both AEMs were yellowish with slight light transmission, as shown in Figure 1C, and have a smooth and dense surface. The QP-1/2 AEM was observed to have good mechanical flexibility. The smooth and dense construction is significant as it inhibits fuel crossover in fuel cells while ensuring excellent dimensional stability [23,24]. Moreover, owing to the uniform and dense distribution of N, the AEMs can combine substantial quantities of OH^−^, providing a favorable structural guarantee for high ionic conductivity in QP-1/x AEMs.

### 2.3. Thermal Stability of sIPN AEMs

**Figure 2:**
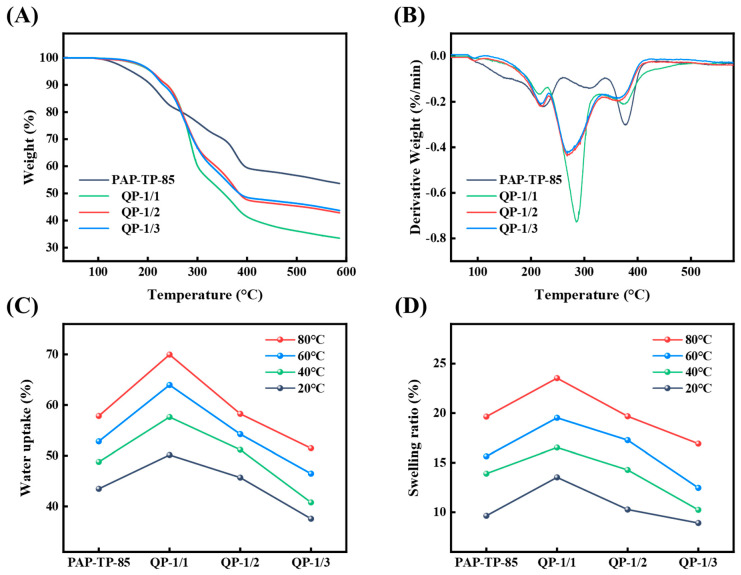
(**A**) The TGA plots of QP-1/x AEMs and PAP-TP-85 AEM. (**B**) The DTG plots of QP-1/x AEMs and PAP-TP-85 AEM. (**C**) Water uptake rate of QP-1/x AEMs and PAP-TP-85 AEM under different temperatures. (**D**) Swelling ratio of QP-1/x AEMs and PAP-TP-85 AEM under different temperatures.

The thermal stability of AEMs is particularly important since AEMFCs operate at temperatures ranging from 60 to 90 °C, requiring good thermal stability of the AEMs. The thermal decomposition temperature (T_d,95_, temperature at 5% weight loss) of the prepared AEMs was investigated. The TGA curves of the OH^−^ form of the QP-1/x AEMs were tested under N_2_ atmosphere (Figure 2A). Before testing, the membrane was treated by holding at 100 °C for 10 min to remove moisture. The PAP-TP-85 was decomposed at T_d,95_ = 167.29 °C, and its weight loss is mainly due to the thermal degradation of the quaternary ammonium group on the molecular chain of PAP-TP-85. The influence of hypercrosslinked polymers QC-DO on the thermal stability of QP-1/x AEMs with semi-interpenetrating network structure is shown in the TGA plot. The thermal stability of the quaternary ammonium groups in QP-1/1, QP-1/2, and QP-1/3 anion exchange membranes is better than that of PAP-TP-85 anion exchange membranes. The QC-DO contains numerous OH^−^ ions that can easily form intramolecular and intermolecular hydrogen bonds, the hydrogen bonding can increase the force between ionic groups and chain segments and improve the thermal stability of ionic polymer. In any case, the thermal degradation temperatures for the AEMs were above the operating temperatures of typical alkaline energy devices.

### 2.4. Water Uptake, Swelling Ratio, and Ion Exchange Capacity (IEC) of sIPN AEMs

AEMs require an appropriate water uptake (WU) and swelling ratio (SR) to ensure efficient OH^−^ conduction. Excessive WU dilutes the concentration of OH^−^, causing a decrease in ionic conductivity of the membrane. High SR compromises the mechanical properties and dimensional stability of AEM. Figure 2C,D demonstrate that the WU and SR of PAP-TP-85 and QP-1/x AEMs are positively associated with the test temperature. As seen in Figure 2, QP-1/2, and QP-1/3 AEMs exhibit appropriate WU and SR; the semi-interpenetrating polymer network plays an important role in maintaining the dimensional stability of membranes. Proper WU and SR promote the generation of hydrated ion phase domains in AEMs that enable efficient ion transport, ensuring good mechanical properties, dimensional stability, and high ionic conductivity.

The IEC values of OH^−^ form QP-1/x AEMs at 25 °C in Table 1. The establishment of semi-interpenetrating networks (sIPN) has been found to be an effective solution to the problem of balancing ionic conductivity and dimensional stability. This is because the increase in the IEC value improves the conductivity of AEMs, but it can adversely affect the alkali stability and mechanical properties of the membrane. Through cross-linking, the IEC value can be increased without negatively impacting the mechanical properties, as the sIPN structure provides additional strength and stability to the membrane.

### 2.5. Mechanical Property and Hydroxide Conductivity of sIPN AEMs

**Figure 3:**
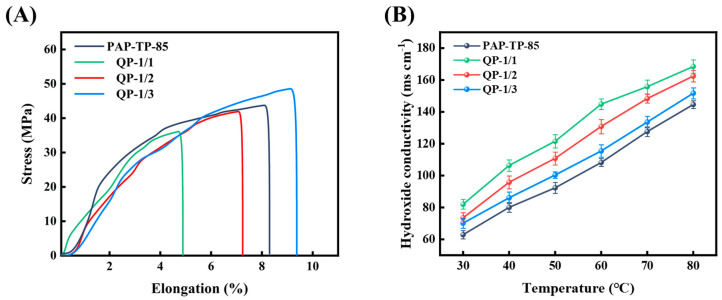
(**A**) Mechanical properties of QP-1/x and PAP-TP-85 AEMs (dry membranes). (**B**) Ionic conductivity plots of OH^−^ form QP-1/x and PAP-TP-85 AEMs at different temperatures.

In the operation of energy devices, AEMs should possess excellent mechanical properties since they are mechanically constrained in between GDLs. Therefore, the mechanical properties of QP-1/x AEMs were evaluated using the fracture tension method. Figure 3A illustrates the mechanical properties of QP-1/x and PAP-TP-85 AEMs. The tensile strength of dry membranes and the elongation at break of membranes tended to increase and then decrease upon the inclusion of QC-DO content in the QP-1/x series membranes, as compared to the linear PAP-TP-85 membranes. This suggests that the increase in cross-linking densities leads to an increase in membrane tension. However, if the cross-linking density exceeds a certain threshold, the intermolecular interactions will become too strong, resulting in increased membrane rigidity and decreased elongation at break. Nonetheless, the QP-1/x AEMs met all the necessary mechanical property requirements required by alkaline energy devices.

The ionic conductivity of hydroxide ions in the QP-1/x AEMs gradually increased with increasing temperature (Figure 3B). In addition, increasing the QC-DO content leads to an increase in the number of quaternary ammonium groups in the AEM. This increase in groups further enhances the ionic conductivity of the membrane. As a result, the ionic conductivity of the composite membrane exhibits a trend of QP-1/1 > QP-1/2 > QP-1/3 > PAP-TP-85. However, it should be noted that the mechanical properties of QP-1/1 AEM are significantly inferior to QP-1/2 AEM. AFM images provided in Figure S5a–d demonstrated that the microphase separation of the prepared QP-1/x AEMs is uniform and distinct. The darker regions represent the hydrophilic parts, whereas the lighter regions are the hydrophobic regions [25]. A more significant and denser microphase separation is observed in the QP-1/2 AEM (Figure S5c), promoting rapid ion transport, and thereby increasing the hydroxide ion conductivity. Overall, the performance of QP-1/x AEMs has certain advantages over some previously reported AEMs [26,27]. These results indicate that QP-1/x AEMs hold the potential for fuel cells and water electrolyzers well.

### 2.6. Chemical Stability of sIPN AEMs

**Figure 4:**
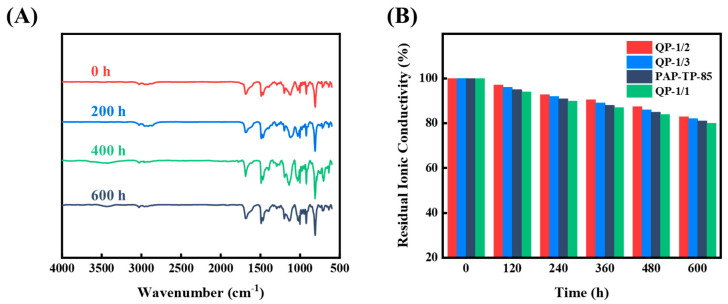
(**A**) FT-IR spectra of QP-1/2 AEM after continuous 600 h alkaline resistance test. (**B**) Residual ionic conductivities of QP-1/x and PAP-TP-85 AEMs after continuous 600 h alkaline resistance test.

The stability of anion exchange membranes (AEMs) under alkaline conditions is a crucial factor for their practical utilization in alkaline energy devices. The degradation mechanisms of AEMs under alkaline conditions involve carbon affinity substitution, Hoffman elimination, and the formation of nitrogen ylides due to the presence of OH^−^ ions [28,29,30]. The ability of AEMs to transfer OH^−^ ions plays a significant role in determining their operational longevity. Therefore, evaluating the resistance to alkali is vital when considering the application of AEMs in fuel cells. Among various AEM options, the QP-1/2 AEM exhibits the highest alkaline stability. Immersion of QP-1/2 AEM in a 2 M NaOH aqueous solution at 80 °C for 600 h did not result in the emergence of new vibrational peaks or the significant disappearance of existing peaks (Figure 4A). Notably, the stretching vibration peak at 3100 cm^−1^, corresponding to the C-H bond on the benzene ring, and the stretching vibration of the C-N bond at 1190 cm^−1^ remained almost unchanged, highlighting the excellent stability of QP-1/2 AEM under alkaline conditions. Additionally, the stretching vibration peak of C-O at 1300–1000 cm^−1^ confirms the successful synthesis of sIPN network structured composite membranes.

The residual ionic conductivity of QP-1/x AEMs after a 600 h continuous alkali resistance test was analyzed showing that the residual ionic conductivities first decrease rapidly and then decrease slowly with increasing alkali treatment time. The increase in the contents of QC-DO result in the deterioration of the alkali resistance of the membranes, as the water-swelling ability of the membrane becomes larger and more OH^−^ can enter AEM with water, increasing the probability of contact between OH^−^ and quaternary amine groups. The residual ionic conductivity was found to be QP-1/2 > QP-1/3 > PAP-TP-85 > QP-1/1. After 600 h of testing, all AEMs had retained more than 80% of their original ionic conductivities, whereas QP-1/2 AEM exhibited the highest residual ionic conductivity of 83% among the tested membranes (Figure 4B). In conclusion, QP-1/2 AEM shows obvious advantages in alkaline stability compared with some reported membranes, indicating that the alkaline stability of QP-1/2 AEM is sufficient for further operation in AEMFCs.

### 2.7. Fuel Cell and Alkaline Water Electrolysis Applications

**Figure 5:**
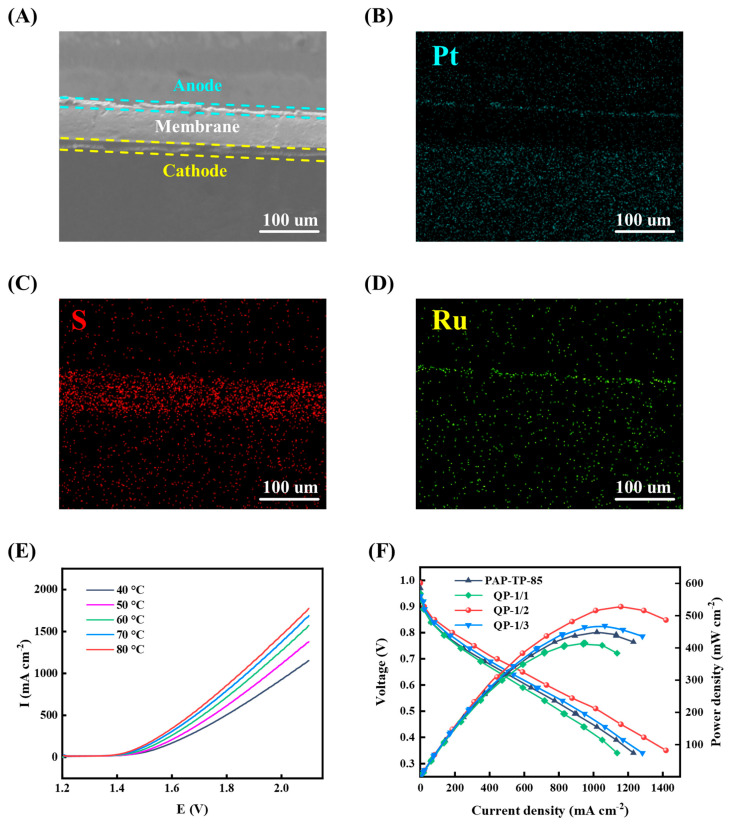
(**A**–**D**) SEM and EDS analysis of the QP-1/2-based MEA cross and surface section. (**E**) Polarization curves for alkaline water electrolysis based on QP-1/2 AEM at 40, 50, 60, 70, and 80 °C and ambient pressure by circulating a 1 M KOH solution in the anode and cathode at a rate of 3 mL min^−1^. (**F**) Polarization curves and power density curves of four AEMs. Testing conditions: 1000/1000 mL min^−1^ A/C flow rate, 80 °C, without backpressure.

For H_2_/O_2_ single-cell testing, MEAs were constructed using QP-1/x AEMs, which were operated without backpressure at a flow rate of 1000/1000 mL min^−1^ A/C and at 80 °C with 100% relative humidified H_2_ as fuel and O_2_ as oxidant. The MEA’s catalyst layer dispersibility was examined using scanning electron microscopy (SEM) and energy dispersive spectrometer (EDS) as shown in Figure 5. Figure 5A reveals that the thicknesses of the cathode and anode catalyst layers were similar with no gaps present between the catalyst layer and AEM, resulting in tight combination. Figure 5B,D show that the catalysts Pt and Ru are uniformly distributed on both sides of the membrane. Figure 5C confirmed the successful construction of the sIPN network by the presence of sulfur elements within the film. 

As depicted in Figure 5E, I–V polarization curves were performed at different temperatures under 1 M KOH. With temperature increase, electrochemical performance is improved. The MEA assembled with QP-1/2 AEM produced the highest current density (1.46 A cm^−2^ at 2.0 V) at 80 °C, which was mainly attributed to the high OH^−^ ion conductivity of QP-1/2 AEM. Durability testing of MEA was carried out under an electrolysis current of 500 Ma cm^−2^ at 60 °C using 1 M KOH fed anode and cathode. Figure S6 shows that during the initial several hours, the voltage of the cell slightly increases, possibly because of the catalyst’s delamination from the catalyst layer [31]. The voltage then remains constant until 9 h, following which it undergoes a slight increase due to light membrane degradation resulting from the OH^−^ attack, which reduces hydroxide conductivity and results in an increased internal resistance. Thereafter, the voltage remains at an average value of 1.82 ± 0.03 V, with minimal fluctuations that can be ascribed to the dynamic nature of water electrolysis. This may arise due to gas accumulation/release from the surface of the catalysts, water uptake changes in the cathode due to water electrolysis and electroosmotic flux. Additionally, water was manually added to the electrolyte solution to sustain the constant KOH concentration, which may cause mild fluctuations. Despite some slight fluctuations, the voltage remains virtually constant over 10 h, indicating the good stability of QP-1/2 AEM-based alkaline water electrolysis.

The AEMs’ exceptional airtightness and gas separation capability is demonstrated by the nearly 1.00 V open circuit voltage (OCV) of all samples [32], as depicted in Figure 5F. The QP-1/2 AEM exhibited the highest peak power density (PPD) of 521 mW cm^−2^ at a current density of 1157.80 mA cm^−2^, which is consistent with previous excellent testing performance, as shown in Figure 5F. Although the PPD of the QP-1/2 AEM is not particularly high when compared to the reported data, single-cell performance depends heavily on the MEA’s preparation method, catalyst, ionomer content, and operating conditions. This makes it challenging to compare QP-1/x AEMs’ single-cell performance with the current literature. Furthermore, this work is a preliminary exploration of QP-1/x AEMs’ feasibility in actual alkaline membrane fuel cell devices, with the MEA preparation and operating conditions not being optimized. The authors anticipate that by optimizing these conditions, QP-1/x AEMs’ single-cell performance can yield even better results.

## 3. Materials and Methods

### 3.1. Materials

Cyclen, 3,6-dioxo-1,8-octanedithiol, 2,2-dimethoxy-1,2-diphenylethanone (DMPA), ethyl ether, isopropanol, and allyl bromide were purchased from Aladdin (Shanghai, China). Potassium carbonate, sodium hydroxide, and ethanol, methanol, and dimethyl sulfoxide (DMSO) were purchased from Sinopharm Chemical Reagent Co. (Shanghai, China). The Pt/C catalyst was supplied by Johnson Matthey with 60 wt.% Pt on a Vulcan XC-72 carbon carrier. Deionized water was utilized and filtered by a Millipore system. All chemicals and solvents used in this study were analytically graded and commercially available and were not further purified before use.

### 3.2. Synthesis of Quaternized Cyclen (QC)

To synthesize quaternized cyclen (QC), the cyclen (1.00 g, 5.80 mmol) and allyl bromide (8.43 g, 69.65 mmol) were dissolved in 60.00 mL of acetonitrile, and then potassium carbonate (3.20 g, 23.15 mmol) was added. The reaction was refluxed at 80 °C for 72 h. After cooling to room temperature, the solid precipitate was filtered off, and then the yellow clarified solution was added dropwise to diethyl ether. The solid product obtained was washed several times with diethyl ether and dried under vacuum at room temperature to obtain a light yellow solid named QC. The synthesis route of QC is illustrated in Scheme S1a.

### 3.3. Synthesis of Hypercrosslinked Polymers by QC

The synthesis of hypercrosslinked polymers by QC was performed using a thiol–ene coupling reaction. Firstly, QC (0.12 mmol, 0.10 g) and 3,6-dioxa-1,8-octanedithiol (0.49 mmol, 0.09 g) were dissolved in DMSO to form a 37.50 wt.% solution. Then, photo-crosslinking was initiated by the addition of DMPA (5 wt.%, 9.45 mg), and the mixture was exposed to a UV light at 365 nm for 2 h. The resulting material was named QC-DO. The synthesis route of QC-DO as shown in Scheme S1b.

### 3.4. Synthesis of the Poly(aryl piperidinium) (PAP) Polymers

The PAP polymers were synthesized in a two-step procedure. The first step involved the synthesis of neutral poly(aryl piperidine) polymers, known as PAP-N. This was carried out using N-methyl-4-piperidone, 2,2,2-trifluoroacetophenone, and p-terphenyl via the method of polyhydroxy alkylation. In the second step, PAP-N was used to synthesize poly(aryl piperidinium) (PAP) by reacting it with iodomethane through the Menshutkin reaction. The synthesis procedure for PAP-N involved dissolving N-methyl-4-piperidone, 2,2,2-trifluoroacetophenone, and p-terphenyl in methylene chloride. To this, 1,1,1-trifluoroacetic acid (TFA) and trifluoromethanesulfonic acid (TFSA) were slowly added dropwise at 0 °C. The reaction was continued at this temperature for 24 h. The resulting solution was then added slowly to an aqueous ethanol solution, and the fibrous solid was filtered, washed, and immersed in 1 M K_2_CO_3_ at 50 °C for 12 h. Finally, the product was filtered, washed, and dried under vacuum at 60 °C, yielding 90% polymer. The synthesis procedure for PAP-TP-85 involved suspending PAP-TP-N-85 in DMSO, followed by adding methyl iodide and stirring the solution for 12 h at room temperature. The solution was then added dropwise into ether, and the solid was filtered, washed, and dried under vacuum at 60 °C, with a yield of almost 90%. PAP-TP-85 represents PAP based on p-terphenyl, with 85 being the molar ratio between N-methyl-4-piperidone and aryl monomers (in percent), and 15 being the molar ratio between 2,2,2-trifluoroacetophenone and aryl monomers (in percent). The synthesis routes for PAP-N and PAP-TP-85 are shown in Scheme S2.

### 3.5. Preparation of QC-DO/PAP-TP-85 Anion Exchange Membranes

To prepare QP-1/x anion exchange membranes with the semi-interpenetrating network, QC-DO and PAP-TP-85 were weighed according to different mass ratios. For convenience, the prepared QC-DO/PAP-TP-85 composite films were labeled as QP-1/x, with 1/x being the mass ratio of QC-DO to PAP-TP-85 in the composite films (1/x = 1:1, 1:2, 1:3). The obtained QC-DO was formulated into a 5 wt.% DMSO dispersion with DMSO solvent, and a 10 wt.% DMSO solution of PAP-TP-85 was dropped into it. In this process, the hypercrosslinked polymers QC-DO dissolved and formed a stable semi-interpenetrating network structure with the linear polymer PAP-TP-85. After the film-forming solution was ultrasonically defoamed and filtered to remove impurities, it was poured onto a glass plate and baked at 60 °C for 12 h. After the dried QC-DO/PAP-TP-85 film was carefully removed from the glass plate, it was immersed in a 1.00 mol L^−1^ NaOH solution for ion exchange to exchange I^−^ to OH^−^. The NaOH solution was changed every 2 h during the ion exchange process, and the completion of ion exchange was detected by HNO_3_ and AgNO_3_. Finally, the QP-1/x membranes were washed in N_2_-saturated deionized water to neutral, cut to the desired shape, and then immersed in N_2_-saturated deionized water for backup.

### 3.6. Characterization

The morphologies of the samples were observed by scanning electron microscopy (SEM, Hitachi S-4700, Tokyo, Japan) fitted with an energy dispersive spectrometer (EDS) detector and via HRTEM (FEI Tecnai G2 F20, Hillsboro, OR, USA). Prior to the test, the samples were sprayed with gold in a vacuum. The infrared spectra were measured on a PerkinElmer-6700 (Glen Waverley, Australia), at a measurement range of 4000–600 cm^−1^ with a resolution of 4 cm^−1^. ^1^H NMR spectra were recorded on a Varian 400 MHz spectrometer using CDCl3, or DMSO-*d*_6_. The thermal stabilities of the AEMs were determined by thermogravimetric analysis (TGA, Universal Analysis 2000) from 30 °C to 800 °C at a heating rate of 10 °C min^−1^ under an N_2_ atmosphere. The stress and elongation of the AEMs were measured with an Instron 5900 system, and the tensile rate was measured at 5 mm min^−1^.

To calculate the water uptake (WU) and swelling ratio (SR) of the AEMs, the weight and length of the wet membranes were determined after wiping the extra water. The AEMs were first dried under vacuum at 80 °C until their weight remained constant. Subsequently, the dried AEMs were soaked in deionized water for 24 h at different temperatures. The test results of the AEMs were then obtained using Equations (1) and (2) to characterize their WU and SR, respectively.

(1) $$\mathrm{WU}\left(\%\right)=\frac{W_{w}-W_{d}}{W_{d}}\times 100\%$$

(2) $$\mathrm{SR}\left(\%\right)=\frac{L_{w}-L_{d}}{L_{d}}\times 100\%$$

Here, W_w_ and W_d_ denote the weights of wet and dried AEMs, while L_w_ and L_d_ denote their respective lengths.

Samples were analyzed using a standard titration approach to determine their ion exchange capacity (IEC). The IEC could be calculated using Equation (3).

(3) $$\mathrm{IEC}=\frac{C(V_{1}-V_{2})}{M_{d}}$$

Here, C represents the concentration of NaOH solution, V_1_ and V_2_ are the volumes of HCl and NaOH solutions used to soak the AEMs and titrate, respectively, and M_d_ is the mass of the dried AEMs. 

To determine the ionic conductivity (σ) of the AEMs, four-probe AC impedance spectroscopy was performed using CHI660C electrochemical equipment with a frequency range of 1 Hz to 1 × 10^6^ Hz. AC impedance measurements were obtained in ultrapure water saturated with N_2_, and the ion conductivity of the membrane (mS cm^−1^) was calculated using Equation (4).

(4) $$\sigma =\frac{L}{\mathrm{RS}}$$

Here, L (cm) represents the distance between the two electrodes, R (kΩ) is the AC impedance of the AEMs, and S (cm^2^) is the AEMs’ cross-sectional area.

The alkaline resistance of the AEMs was evaluated by measuring changes in ionic conductivity before and after immersion in 1 M NaOH solution at 60 °C, over a total test time of 600 h (measured every 120 h at 80 °C).

The performance of the QP-1/2 AEM for water electrolysis was also considered. Pt/C and NiCoFe were used as the cathode catalyst and the anode catalyst, respectively. The catalyst loadings were controlled at 0.5 mg cm^−2^ for both the electrodes and the active area was 1 cm^−2^. The MEA prepared by the hot-pressed method was assembled into a water electrolysis cell. The polarization curve was collected at 40, 50, 60, 70, and 80 °C by the voltage sweep method, and the potential was increased at a rate of 5 mV s^−1^. The durability was evaluated at 60 °C by feeding a 1 M aqueous KOH solution into both the electrodes at a flow rate of 3 mL min^−1^ and applying a constant current density of 500 mA cm^−2^.

The catalyst ink was prepared by mixing Pt/C catalyst (cathode, 60 wt.%, Johnson Matthey) or PtRu/C (anode, 40 wt.% of Pt and 20 wt.% of Ru), ionomer (5 wt.% in DMSO), water, and isopropanol at a concentration of 3 wt.%. The ratio of catalyst ionomer was set to 4:1. The PAP-BP-100 ionomer was used for the anode and cathode catalyst layers [14]. After being sonicated for 3 h while keeping the bath temperature lower than 5 °C, the ink was sprayed on the cathode and anode of the AEM to obtain a catalyst-coated membrane (CCM). The noble metal loadings at the cathode and anode were 0.5 mg cm^−2^ Pt and 0.5 mg cm^−2^ Pt/Ru, respectively. Finally, fuel cell performance was measured on a Fuel Cell Test System under 100% RH at 80 °C, and the flow rate of H_2_/O_2_ was 1 L min^−1^ without backpressure.

## 4. Conclusions

A series of QP-1/x anion exchange membranes (AEMs) was prepared by incorporating hypercrosslinked polymers QC-DO as the framework material and PAP-TP-85 as the primary anion carrier. The introduction of QC-DO in AEMs resulted in apparent microphase separation within the membranes. The semi-interpenetrating polymer network guarantees satisfying mechanical properties and dimensional endurance, as well as high ionic conductivity. The obtained QP-1/x AEMs were proven to be smooth and tough, demonstrating the positive effect of hypercrosslinked polymers QC-DO on the mechanical properties of the AEM. The enhanced alkaline stability is noteworthy due to the effective limitation of hydroxide attack by the semi-interpenetrating network. Notably, the ionic conductivity and fuel cell peak power density of QP-1/2 AEMs were measured to be 162.5 mS cm^−1^ and 521 mW cm^−2^. Moreover, alkaline water electrolyzers based on QP-1/2 AEM demonstrated a current density of 1460 mA cm^−2^. The QP-1/x AEMs with the semi-interpenetrating network have great potential for alkaline membrane fuel cells and water electrolyzers.

## Figures and Tables

**Table 1:** IEC values of OH^−^.

Samples	Theoretical IEC	Titration IEC
PAP-TP-85	2.35	2.29
QP-1/1	2.72	2.62
QP-1/2	2.60	2.47
QP-1/3	2.54	2.44

## Data Availability

All the data obtained and involved in the research are published in this paper.

## Supplementary Materials

The following supporting information can be downloaded at: https://www.mdpi.com/article/10.3390/molecules28145470/s1, Scheme S1: (a) The synthesis route of QC. (b) The synthesis route of QC-DO; Scheme S2: The synthesis route of PAP-N and PAP-TP-85; Figure S1: ^1^H NMR spectra of QC using CDCl_3_ as a solvent; Figure S2: ^1^H NMR spectra of PAP-N. The spectra were recorded with DMSO-*d*_6_ solutions containing 3–6 vol% of TFA; Figure S3: ^1^H NMR spectra of PTP-TP-85. The spectra were recorded with DMSO-*d*_6_ solutions containing 3–6 vol% of TFA; Figure S4: FT-IR spectroscopy of QC and QC-DO; Figure S5: AFM phase images of (a) PAP-TP-85 AEM, (b) QP-1/1 AEM, (c) QP-1/2 AEM, and (d) QP-1/3 AEM; Figure S6: Long-term durability of the AEMWE voltage during galvanostatic. Experimental conditions used: circulating electrolyte in the anode and cathode 1 M KOH solution, flow rate 3 mL min^−1^, temperature 60 °C, current density 500 mA cm^−2^.

## Sample Availability

Samples of the compounds are not available from the authors upon request.

## References

[1] Kanchanakul I., Srinophakun T.R., Kuboon S., Kaneko H., Kraithong W., Miyauchi M., Yamaguchi A. (2023). Development of Photothermal Catalyst from Biomass Ash (Bagasse) for Hydrogen Production via Dry Reforming of Methane (DRM): An Experimental Study. Molecules.

[2] Vijayakumar V., Nam S.Y. (2019). Recent advancements in applications of alkaline anion exchange membranes for polymer electrolyte fuel cells. J. Ind. Eng. Chem..

[3] Chen N., Lee Y.M. (2021). Anion exchange polyelectrolytes for membranes and ionomers. Prog. Polym. Sci..

[4] Xue J., Zhang J., Liu X., Huang T., Jiang H., Yin Y., Qin Y., Guiver M.D. (2022). Toward alkaline-stable anion exchange membranes in fuel cells: Cycloaliphatic quaternary ammonium-based anion conductors. Electrochem. Energy Rev..

[5] Aili D., Wright A.G., Kraglund M.R., Jankova K., Holdcroft S., Jensen J.O. (2017). Towards a stable ion-solvating polymer electrolyte for advanced alkaline water electrolysis. J. Mater. Chem. A.

[6] Park E.J., Arges C.G., Xu H., Kim Y.S. (2022). Membrane Strategies for Water Electrolysis. ACS Energy Lett..

[7] Chand K., Paladino O. (2023). Recent developments of membranes and electrocatalysts for the hydrogen production by anion exchange membrane water electrolysers: A review. Arab. J. Chem..

[8] Xue B., Wang Q., Zheng J., Li S., Zhang S. (2020). Bi-guanidinium-based crosslinked anion exchange membranes: Synthesis, characterization, and properties. J. Membr. Sci..

[9] Liu F., Yang Q., Gao X., Wu H., Zhang Q., Zhu A., Liu Q. (2020). Anion exchange membranes with dense N-spirocyclic cations as side-chain. J. Membr. Sci..

[10] Karibayev M., Kalybekkyzy S., Wang Y., Mentbayeva A. (2022). Molecular Modeling in Anion Exchange Membrane Research: A Brief Review of Recent Applications. Molecules.

[11] Chen J., Choo Y.S.L., Wang X., Liu Y., Yue X., Gao X., Gao W., Zhang Q., Zhu A., Liu Q. (2023). Effects of the crown ether cavity on the performance of anion exchange membranes. J. Colloid Interface Sci..

[12] Yang Z., Zhang M., Zhao Z., Zhang X., Fan M. (2022). Construction of Quaternized Polysulfone/Polyquaternium-10 Anion Exchange Membrane with Semi-Interpenetrating Network for Alkaline Fuel Cell. Macromol. Mater. Eng..

[13] You W., Noonan K.J.T., Coates G.W. (2020). Alkaline-stable anion exchange membranes: A review of synthetic approaches. Prog. Polym. Sci..

[14] Wang J., Zhao Y., Setzler B.P., Rojas-Carbonell S., Ben Yehuda C., Amel A., Page M., Wang L., Hu K., Shi L. (2019). Poly(aryl piperidinium) membranes and ionomers for hydroxide exchange membrane fuel cells. Nat. Energy.

[15] Chen N., Hu C., Wang H.H., Kim S.P., Kim H.M., Lee W.H., Bae J.Y., Park J.H., Lee Y.M. (2021). Poly(Alkyl-Terphenyl Piperidinium) Ionomers and Membranes with an Outstanding Alkaline-Membrane Fuel-Cell Performance of 2.58 W cm^−2^. Angew. Chem. Int. Ed. Engl..

[16] Olsson J.S., Pham T.H., Jannasch P. (2018). Poly(arylene piperidinium) Hydroxide Ion Exchange Membranes: Synthesis, Alkaline Stability, and Conductivity. Adv. Funct. Mater..

[17] Dong D., Xiao Y., Zhang M., Yang Z., Wang K., Fan M. (2022). Crosslinked anion exchange membranes with regional intensive ion clusters prepared from quaternized branched polyethyleneimine/quaternized polysulfone. Int. J. Hydrogen Energy.

[18] Dong D., Zhang M., Xiao Y., Yang Z., Wang K., Fan M. (2022). Quaternized Branched Polyethyleneimine Modified Nitrogen-Doped Graphene Quantum Dots/Quaternized Polysulfone Composite Anion Exchange Membranes with Improved Performance. Macromol. Mater. Eng..

[19] Zhang S., Wang Y., Liu P., Wang X., Zhu X. (2020). Photo-cross-linked poly(N-allylisatin biphenyl)-co-poly(alkylene biphenyl)s with pendant N-cyclic quaternary ammonium as anion exchange membranes for direct borohydride/hydrogen peroxide fuel cells. React. Funct. Polym..

[20] Chen W., Shen H., Gong Y., Li P., Cheng C. (2023). Anion exchange membranes with efficient acid recovery obtained by quaternized poly epichlorohydrin and polyvinyl alcohol during diffusion dialysis. J. Membr. Sci..

[21] Gong Y., Chen W., Shen H., Cheng C. (2023). Semi-interpenetrating Polymer-Network Anion Exchange Membrane Based on Quaternized Polyepichlorohydrin and Polyvinyl Alcohol for Acid Recovery by Diffusion Dialysis. Ind. Eng. Chem. Res..

[22] Zhu L., Zimudzi T.J., Li N., Pan J., Lin B., Hickner M.A. (2016). Crosslinking of comb-shaped polymer anion exchange membranes via thiol–ene click chemistry. Polym. Chem..

[23] Niu M., Zhang C., He G., Zhang F., Wu X. (2019). Pendent piperidinium-functionalized blend anion exchange membrane for fuel cell application. Int. J. Hydrogen Energy.

[24] Zhou Y., Zhou L., Feng C., Wu X., Bao R., Liu Z., Yang M., Yang W. (2019). Direct modification of polyketone resin for anion exchange membrane of alkaline fuel cells. J. Colloid Interface Sci..

[25] Zhao Z., Yang Z., Zhang M., Du W., Lan W., Zhang X., Fan M. (2023). Tripartite Cationic Interpenetrating Polymer Network Anion Exchange Membranes for Fuel Cells. ACS Appl. Energy Mater..

[26] Yang Q., Lin C., Liu F., Li L., Zhang Q., Zhu A., Liu Q. (2018). Poly (2,6-dimethyl-1,4-phenylene oxide)/ionic liquid functionalized graphene oxide anion exchange membranes for fuel cells. J. Membr. Sci..

[27] Li J., Wang S., Liu F., Wang X., Chen H., Mao T., Wang Z. (2019). Poly (aryl ether ketone)/polymeric ionic liquid with anisotropic swelling behavior for anion exchange membranes. J. Membr. Sci..

[28] Zhao T., Long C., Wang Z., Zhu H. (2021). Multication Cross-Linked Poly(p-terphenyl isatin) Anion Exchange Membranes for Fuel Cells: Effect of Cross-Linker Length on Membrane Performance. ACS Appl. Energy Mater..

[29] Zeng L., Yuan W., Ma X., He Q., Zhang L., Wang J., Wei Z. (2022). Dual-Cation Interpenetrating Polymer Network Anion Exchange Membrane for Fuel Cells and Water Electrolyzers. Macromolecules.

[30] Dong J., Li H., Ren X., Che X., Yang J., Aili D. (2019). Anion exchange membranes of bis-imidazolium cation crosslinked poly(2,6-dimethyl-1,4-phenylene oxide) with enhanced alkaline stability. Int. J. Hydrogen Energy.

[31] Li G., Chuang P.-Y.A. (2018). Identifying the forefront of electrocatalytic oxygen evolution reaction: Electronic double layer. Appl. Catal. B.

[32] Gao W., Gao X., Choo Y., Wang J., Cai Z., Zhang Q., Zhu A., Liu Q. (2023). Durable dual-methylpiperidinium crosslinked poly(binaphthyl-co-terphenyl piperidinium) anion exchange membranes with high ion transport and electrochemical performance. Chem. Eng. J..

