# Supplementary material for: Semi-Interpenetrating Network Anion Exchange Membranes by Thiol–Ene Coupling Reaction for Alkaline Fuel Cells and Water Electrolyzers
Source: Molecules. 2023 Jul 17;28(14):5470. doi: 10.3390/molecules28145470 (PMC10385286; doi:10.3390/molecules28145470)
Supplement: Supplementary file 1 [file molecules-28-05470-s001.zip › molecules-2510872-supplementary.pdf]

Supplementary Informations

Semi-Interpenetrating Network Anion Exchange Membranes by Thiol–Ene Coupling Reaction for Alkaline Fuel Cells and Water Electrolyzers

Zhiyu Jin, Xiuyang Zou, Guodong Xu, Zhe Sun * and Feng Yan *

Jiangsu Engineering Laboratory of Novel Functional Polymeric Materials, Jiangsu Key Laboratory of Advanced Negative Carbon Technologies, Suzhou Key Laboratory of Soft Material and New Energy, College of Chemistry, Chemical Engineering and Materials Science, Soochow University, Suzhou 215123, China; 20204209321@stu.suda.edu.cn (Z.J.); 20204009045@stu.suda.edu.cn (X.Z.); xuguodong19@163.com (G.X.)

* Correspondence: zsun@suda.edu.cn (Z.S.); fyan@suda.edu.cn (F.Y.)

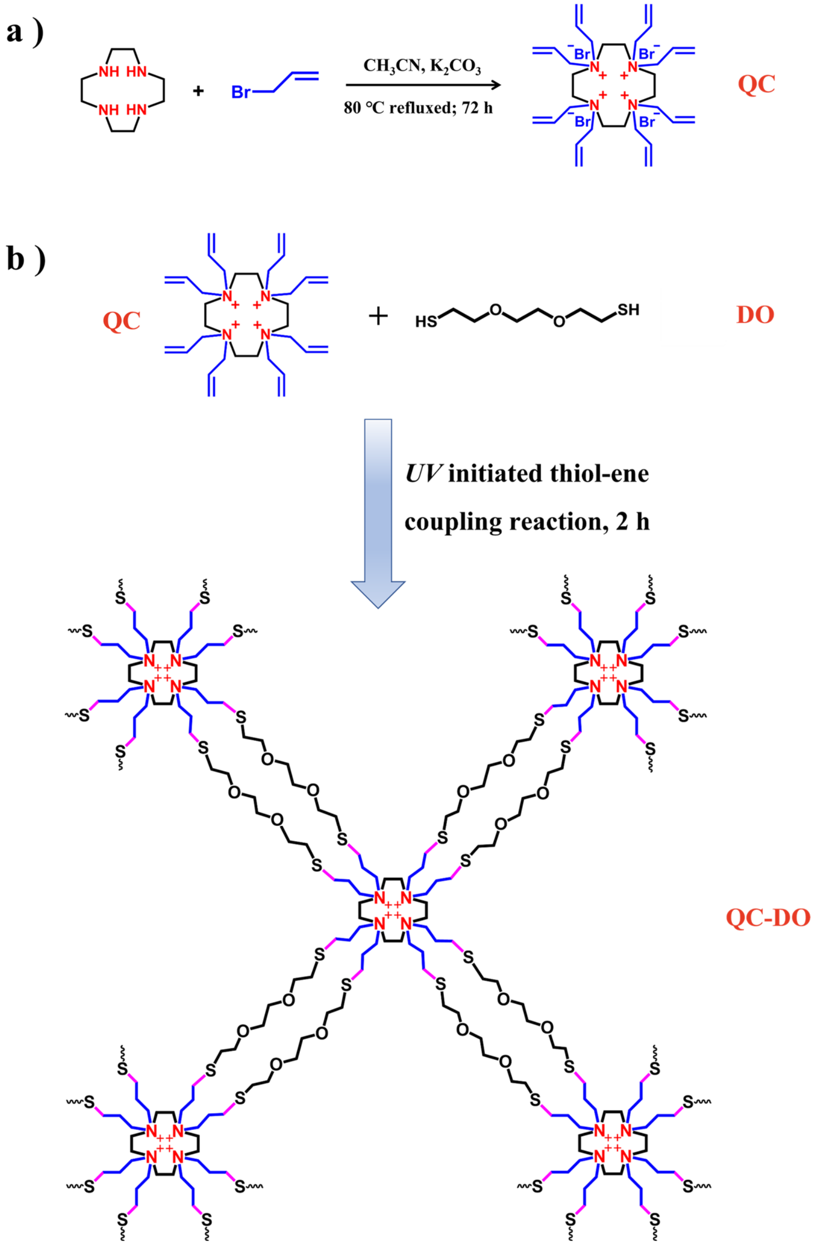

Scheme S1. (a) The synthesis route of QC. (b) The synthesis route of QC-DO.

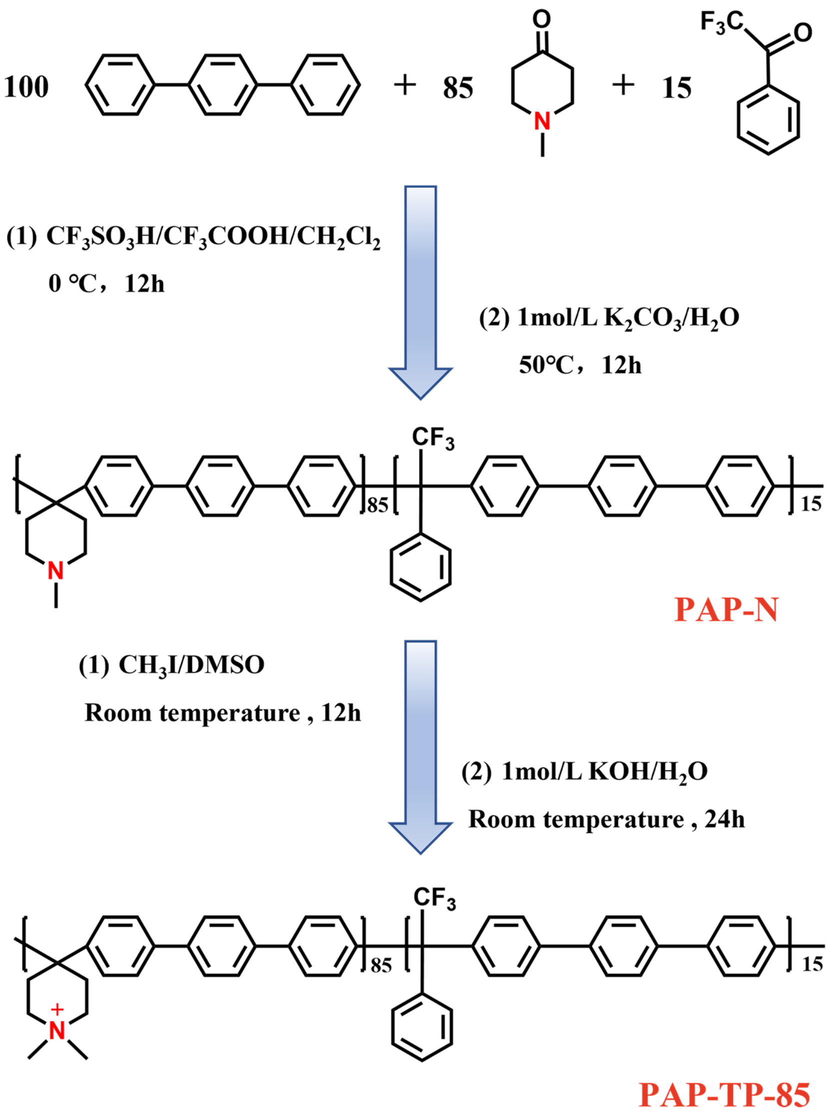

Scheme S2. The synthesis route of PAP-N and PAP-TP-85.

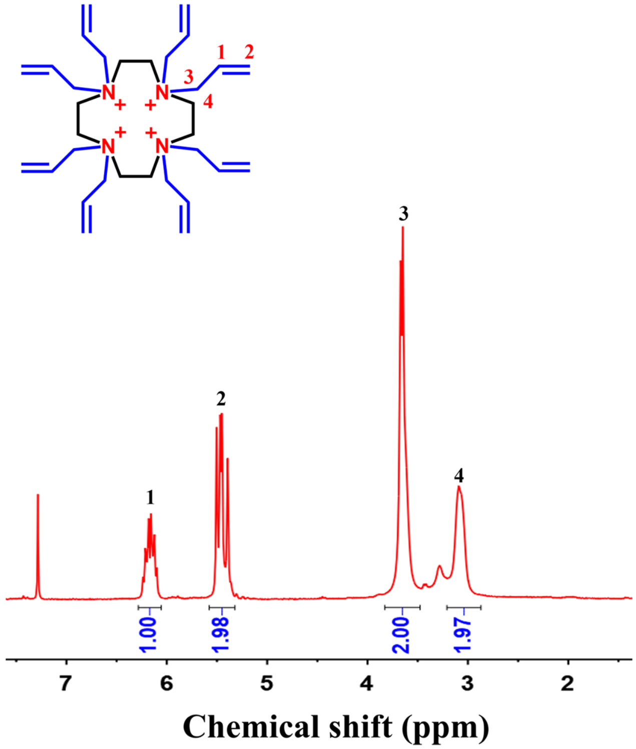

Figure S1. ^{1}H NMR spectra of QC using $CDCl_3$ as a solvent.

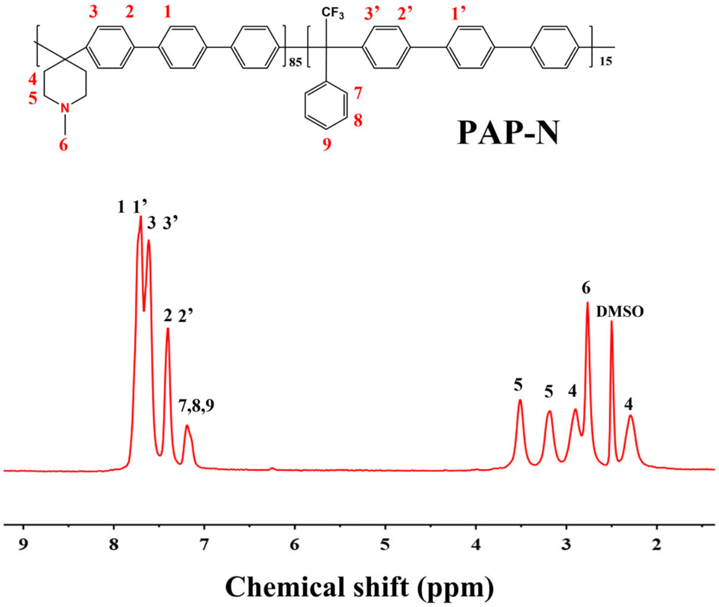

Figure S2. ^{1}H NMR spectra of PAP-N. The spectra were recorded with DMSO-d_6 solutions containing 3-6 vol% of TFA.

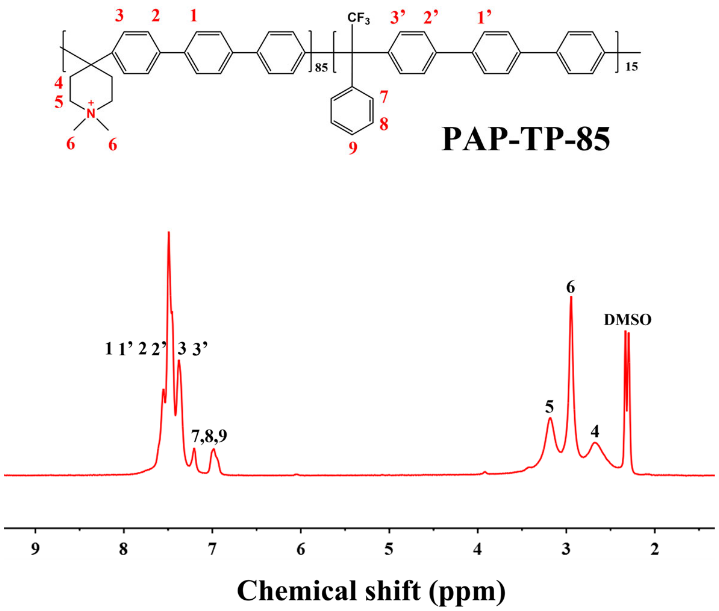

Figure S3. ^{1}H NMR spectra of PTP-TP-85. The spectra were recorded with DMSO-d_6 solutions containing 3-6 vol% of TFA.

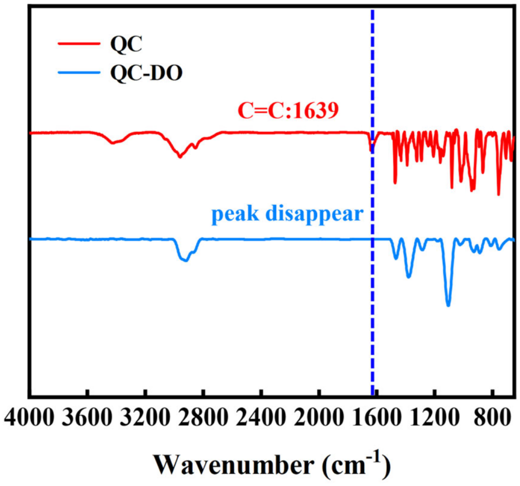

Figure S4. FT-IR spectroscopy of QC and QC-DO.

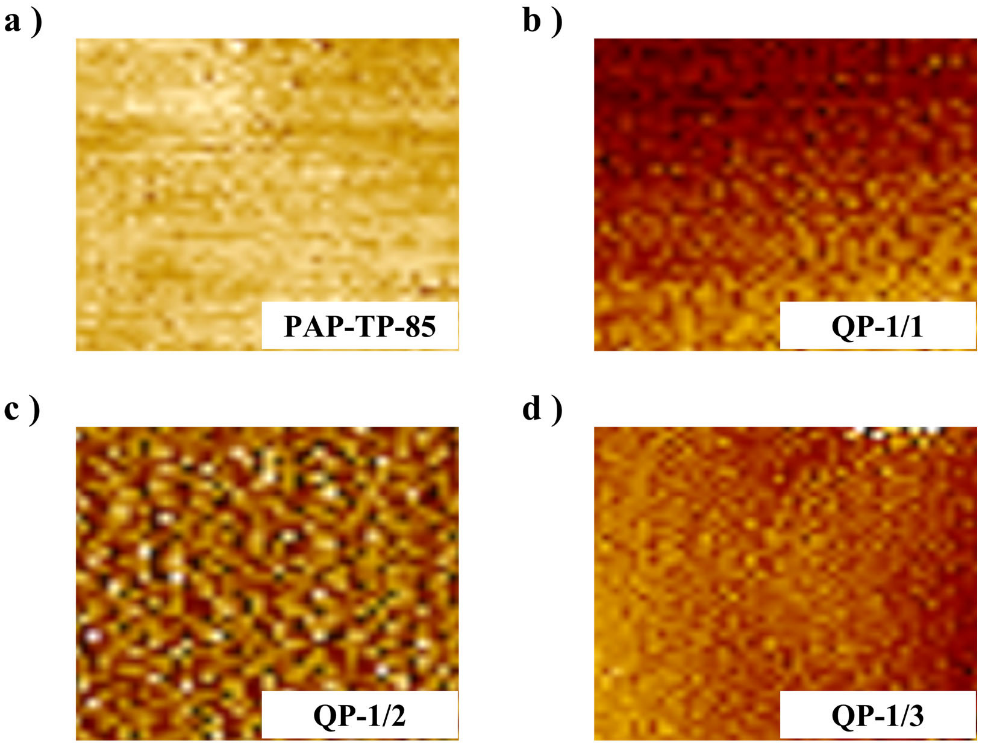

Figure S5. AFM phase images of (a) PAP-TP-85 AEM, (b) QP-1/1 AEM, (c) QP-1/2 AEM, and (d) QP-1/3 AEM.

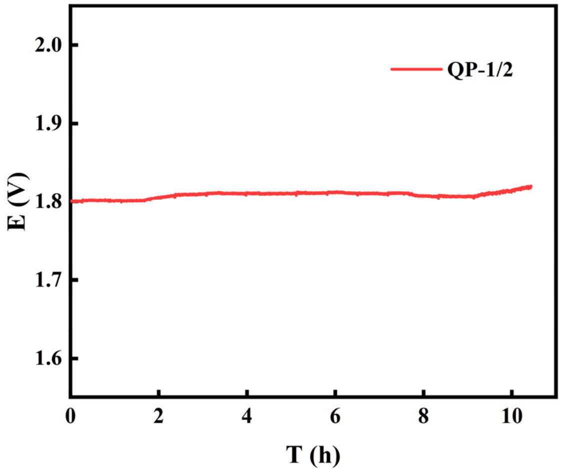

Figure S6. Long-term durability of the AEMWE voltage during galvanostatic. Experimental conditions used: circulating electrolyte in the anode and cathode 1 M KOH solution; flow rate 3 mL min^{-1}; temperature 60 °C; current density 500 mA cm^{-2}.
